# Artificial intelligence for diffusion MRI-based tissue microstructure estimation in the human brain: an overview

**DOI:** 10.3389/fneur.2023.1168833

**Published:** 2023-04-21

**Authors:** Abrar Faiyaz, Marvin M. Doyley, Giovanni Schifitto, Md Nasir Uddin

**Affiliations:** ^1^Department of Electrical and Computer Engineering, University of Rochester, Rochester, NY, United States; ^2^Department of Imaging Sciences, University of Rochester, Rochester, NY, United States; ^3^Department of Biomedical Engineering, University of Rochester, Rochester, NY, United States; ^4^Department of Neurology, University of Rochester, Rochester, NY, United States

**Keywords:** artificial intelligence, machine learning, deep learning, diffusion MRI (dMRI), neuroimaging, brain, microstructure, biophysical model

## Abstract

Artificial intelligence (AI) has made significant advances in the field of diffusion magnetic resonance imaging (dMRI) and other neuroimaging modalities. These techniques have been applied to various areas such as image reconstruction, denoising, detecting and removing artifacts, segmentation, tissue microstructure modeling, brain connectivity analysis, and diagnosis support. State-of-the-art AI algorithms have the potential to leverage optimization techniques in dMRI to advance sensitivity and inference through biophysical models. While the use of AI in brain microstructures has the potential to revolutionize the way we study the brain and understand brain disorders, we need to be aware of the pitfalls and emerging best practices that can further advance this field. Additionally, since dMRI scans rely on sampling of the q-space geometry, it leaves room for creativity in data engineering in such a way that it maximizes the prior inference. Utilization of the inherent geometry has been shown to improve general inference quality and might be more reliable in identifying pathological differences. We acknowledge and classify AI-based approaches for dMRI using these unifying characteristics. This article also highlighted and reviewed general practices and pitfalls involving tissue microstructure estimation through data-driven techniques and provided directions for building on them.

## 1. Introduction

The microstructural estimation of biological tissue through histological analysis is reliable. However, it has limitations, including its invasive nature (1). In contrast, diffusion MRI (dMRI) is a non-invasive technique for encoding information about tissue structure at the microscopic scale in the human brain (2–4). It is based on the restricted diffusion of water molecules in the local microstructural environment. Diffusion tensor imaging (DTI), a widely used dMRI approach, has been shown to be sensitive to pathological changes in the brain (5). However, water diffusion in DTI has been assumed to be a Gaussian process, and microscopic inspection of the neuronal environment invalidated this assumption (6, 7). Thus, DTI is not specific to microstructural properties such as cell size, axon diameter, orientation dispersion, and neurite density (8).

Consequently, multi-compartment modeling in dMRI has emerged as a powerful non-Gaussian tool for studying neuropathogenesis and holds potential for both research and clinical applications, including providing insight into the biological mechanisms of disease and improving diagnosis and treatment monitoring (9). Models such as free water imaging (FWI) and Neurite Orientation Dispersion and Density Imaging (NODDI) helped early multicompartmental modeling become more widely adopted in the field (10, 11). However, most of the advanced multi-compartment dMRI models suffer estimation errors due to highly non-linear signal representations, underlying simplifying assumptions, and sometimes motion artifacts due to longer acquisition times for multiple b-values and diffusion gradient directions (8, 12).

Artificial intelligence (AI) commonly involves creating systems capable of tasks requiring human senses and intelligence, such as speech, image, and natural language synthesis. Deep/machine learning (DL/ML) subsets of AI train deep artificial neural networks to make predictions or decisions from data in a common gradient update framework. AI algorithms have evolved with DL/ML techniques due to skyrocketing computing power and resources (13, 14). Recently, AI-based approaches have been proposed to address issues such as denoising and artifact reduction (15–18), fiber tractography (19–21), resolution enhancement (22–25), and quantification of microstructural properties (20–22, 26–31). These techniques have been particularly useful for working with clinical and challenging datasets and have led to advances in dMRI parameter mapping and image quality assessment and improvement. With that said, it is important to understand that there are potential pitfalls of using such data-driven techniques, e.g., tomographic hallucination, training bias, etc. (32–34); thus, the application of such an approach in clinical dMRI needs to follow the best practices; otherwise, it can lead to inaccurate or unreliable results, proving itself to be a double-edged sword. Best practices for AI in clinical dMRI include proper protocol design and optimization, accurate image acquisition and noise-redacted reconstruction for standardized ground truth, and careful interpretation of the data (13). Further, efforts are currently being made to quantitatively understand how much clinically relevant information can be retrieved through DL/ML architectures, which is another important aspect of clinical dMRI besides general image/parameter reconstruction (35).

This study briefly generalized common features related to some of the leading biophysical models that have practically established sensitivity to the designed parameters for the models and mainly reviewed the concurrent AI alternatives to these models with their general architecture/results to probe the best practices. This article also discussed the challenges of existing AI approaches and future perspectives in tissue microstructure estimation. We identified the gross development of these algorithms in the targeted microarchitecture modeling. This includes highlighting efforts/innovations in data engineering, feature design, common practices in these techniques, or differences that set the methods apart for better/worse results.

## 2. Biophysical models of dMRI

### 2.1. The domain of dMRI sensitivity in biophysical models

In dMRI techniques, diffusion-weighted (DW) images are acquired with multiple b-values at a different number of gradient directions (2, 36–38). Then, microstructure information in each voxel of the image can be extracted via either *signal representations* (e.g., DTI, diffusion kurtosis imaging DKI, fiber orientation distribution function fODF) or *biophysical models* (e.g., NODDI, spherical mean technique SMT, white matter tissue integrity WMTI) (5, 39–41).

Signal representations explain the DW signal behavior and provide effective summary statistics at a given voxel that do not rely on assumptions about the underlying tissue properties, which is clinically demanding but estimated parameters lack specificity. On the other hand, biophysical models are mathematical models aiming to explain the physical properties of biological systems. The parameters of the biophysical models in dMRI are intentionally designed to be adjustable, mimicking the biological constructs to fit the measured DW signals. These constructs may include biophysically meaningful parameters such as tissue volume fractions and other properties that are specific to the system being studied (40). For example, white matter models vastly differ from gray matter models, leading to differentiated assumptions and geometrical functions (42, 43). A detailed explanation of the steps involved in constructing a biophysical model for dMRI can be found in Jelescu et al. (9).

The advent of computational power, complex mathematics, and geometrical formulations has led to a good number of biophysical models of brain tissue to date, including the standard model, SMT, AxCaliber (44), diffusion basis spectrum imaging (DBSI) (45), NODDI (11), WMTI (46), simple harmonic oscillator-based reconstruction and estimation (SHORE) (47) and soma and neurite density imaging (SANDI) (48). Biophysical models with their DL/ML alternatives/improvements found in the past decade are considered within the scope of this study and are displayed in Table 1 Block-A and Supplementary Table S1.

**Table 1:** Summary of biophysical models with their key parameters and studies conducted on diffusion MRI using AI in human brain.

**Block A: Biophysical models of dMRI**				**Block B: AI agnostic to q-space geometry**			
**Year**	**Biophysical Models**	**Protocol feasibility**	**Key parameters/ scalars**	**Year**	**AI Models**	**General architecture**	**Task**
2009	FWI (10)	Single/Multishell	FW, FWE-DT	2022	AEME (75)	LSTM	NODDI
2012	NODDI (11)	Multishell	NDI, ODI, f_ISO_ (or FW)	2022	METSC (Adapted from ViT) (31)	Transformer (Encoder- Decoder)	NODDI
2015	SHORE (47)	Multishell	RTOP, MSD	2022	SDnDTI (17)	Modified U-net	DTI denoising
2016	SMT (12, 76)	Multishell	*FA*_*SMT*_, *MD*_*SMT*_, λ_⊥_, λ_||_	2022	Transformer (77)	Transformer (Attention)	DTI
2018	Standard model (78)	Multishell	*f*_*i*n_, *D*_*a*_, $D_{e}^{⊥}$, $D_{e}^{\left|\right|}$	2022	ADL (Atlas powered DL) (79)	U-net++	FA, ODI
2020	SANDI (Ball, Stick, Sphere) (48)	Multishell (b-value > 3,000 s/mm^2^)	*f*_*in*_, *f*_*ec*_, *f*_*is*_, *D*_*in*_, *D*_*ec*_, *r*_*s*_	2022	VRfRNet^*^ (21)	GAN	fODF
**Block C: AI with active use of q-space geometry**				2021	IQT with Auto-Encoder (80)	Residual Network	DTI SR
**Year**	**Models**	**General architecture**	**Task**	2021	SRDTI (16)	CNN (3D)	DTI SR
2023	ED-RNN (67)	RNN based encoder- decoder	WMTI-Watson	2021	Multimodal SRqDL (23)	CNN (3D)	NODDI, SMT SR
2022	HGT (based on TAGCN (81) + RDT) (24)	Two different stages: GCN and Transformer (Attention)	NODDI	2021	Super resolved q-space DL (SRqDL) (74)	CNN	NODDI
2022	HemiHex-MLP (68)	Adapted MLP	DTI	2021	SuperDTI (82)	U-Net	DTI
2021	Spherical CNN^**^ (29)	CNN	NODDI Super angular resolution	2021	Fetal MRI (18)	CNN + Residual Block	DTI
2021	Bottleneck DL^*^ (28) Adapted SHResNet and M-heads (73, 83)	CNN + Residual Block	DTI, Ball and Stick, IVIM, SMT, NODDI	2020	DeepDTI (15)	CNN	DTI
2021	q-space feature-based MLP (20)	MLP	fODF	2019	Bayesian (71)	Bayesian ML	Any, NODDI
2021	q-space conditioned DWI Generator (84)	U-Net, GAN	NODDI, SHORE, DKI, fODF	2019	SHResNet (73)	CNN + Residual Block	DWI harmonization
2020	GCNN (85)	GCN	NODDI	2019	MESC-Net (27)	LSTM	SMT, NODDI, SHORE
2019	CNN^*^ (19)	CNN (3D)	fODF	2019	CNN-NODDI (26)	CNN	NODDI, GFA
**Block D: Models leveraging AI and Maximum Likelihood**				2018	Deeper IQT with RevNet (86)	ML	DTI SR
**Estimation (MLE) frameworks**						
**Year**	**Recent trends in AI models**	**AI-MLE Integrated architecture**	**Task**	2017	MEDN/MEDN+ (51)	Adapted MLP	NODDI
2021/2022	DL prior NODDI (30, 72)	Modified MLP initializes MLE	Single Shell NODDI	2017	IQT (52)	ML (Regression Forest)	NODDI, SMT
2022	DL-MLE (58)	Modified MLP initializes MLE	NODDI	2017	Trained Random Forest (87)	ML (Regression Forest)	Permeability
2023	dtiRIM (54)	Modified RNN calculating MLE gradient	DTI	2016	q-DL (22)	MLP	DKI, NODDI

Tasks indicate the parameters of models on which the learning algorithms have been applied. Parameters refer to micro-parameters of different models of diffusion signal. ^*^ indicates spherical harmonics (SH) coefficients are used as network input, not the DWIs directly. ^**^ indicates convolutional features are extracted on spherical (S^2^) domain. FWI, Free Water Imaging; FW, Free Water; FWE-DT, Free Water Eliminated Diffusion Tensor; NODDI, Neurite Orientation and Dispersion Imaging; NDI, Neurite Density Index; ODI, Orientation Dispersion Index; f_ISO_, isotropic volume fraction; SHORE, Simple Harmonic Oscillator-based Reconstruction and Estimation; RTOP, Return-To-Origin Probability; MSD, Mean Squared Displacement; SMT, Spherical Mean Technique; FA, Fractional Anisotropy; GFA, generalized FA; MD, Mean Diffusivity; λ_||_, λ_⊥_, Longitudinal and Transverse Diffusivity Constants; D${}_{\text{e}}^{⊥}$, D${}_{\text{e}}^{\left|\right|}$, Extracellular Diffusion coefficients; D_a_, *D*_*in*_, Intracellular Diffusion coefficients; *f*_*in*_, *f*_*ec*_, *f*_*is*_, intracellular, extracellular and free volume fractions; SANDI, Soma And Neurite Density Imaging; AEME, adaptive network with extragradient for diffusion MRI-based microstructure estimation; METSC, Microstructure Estimation Transformer with Sparse Coding; SDnDTI, Self-supervised deep learning-based denoising for DTI; VRfRNet, Volumetric ROI fODF reconstruction network; IQT, image quality transfer; CNN, convolutional neural network; RNN, Recurrent Neural Network; ED-RNN, Encoder Decoder RNN; HGT, Hybrid Graph Transformer; TAGCN, Topology Adaptive Graph Convolutional Network; RDT, Residual Dense Transformer; MLP, Multi-Layered Perceptron; GAN, Generative Adversarial Network; LSTM, Long Short-Term Memory; SHResNet, Spherical Harmonics Residual network; WMTI, White Matter Tract Integrity; SR, Super Resolution; DKI, Diffusion Kurtosis Imaging; GCN, Graph Convolution Network; GCNN, graph convolutional neural network; IVIM, intravoxel incoherent motion; fODF, fiber orientation distribution function; MEDN, Microstructure Estimation using a Deep Network; q-DL, q-space deep learning; dtiRIM, DTI with recurrent inference machines.

### 2.2. Parametrizations, estimation, and validation

**Figure 1:**
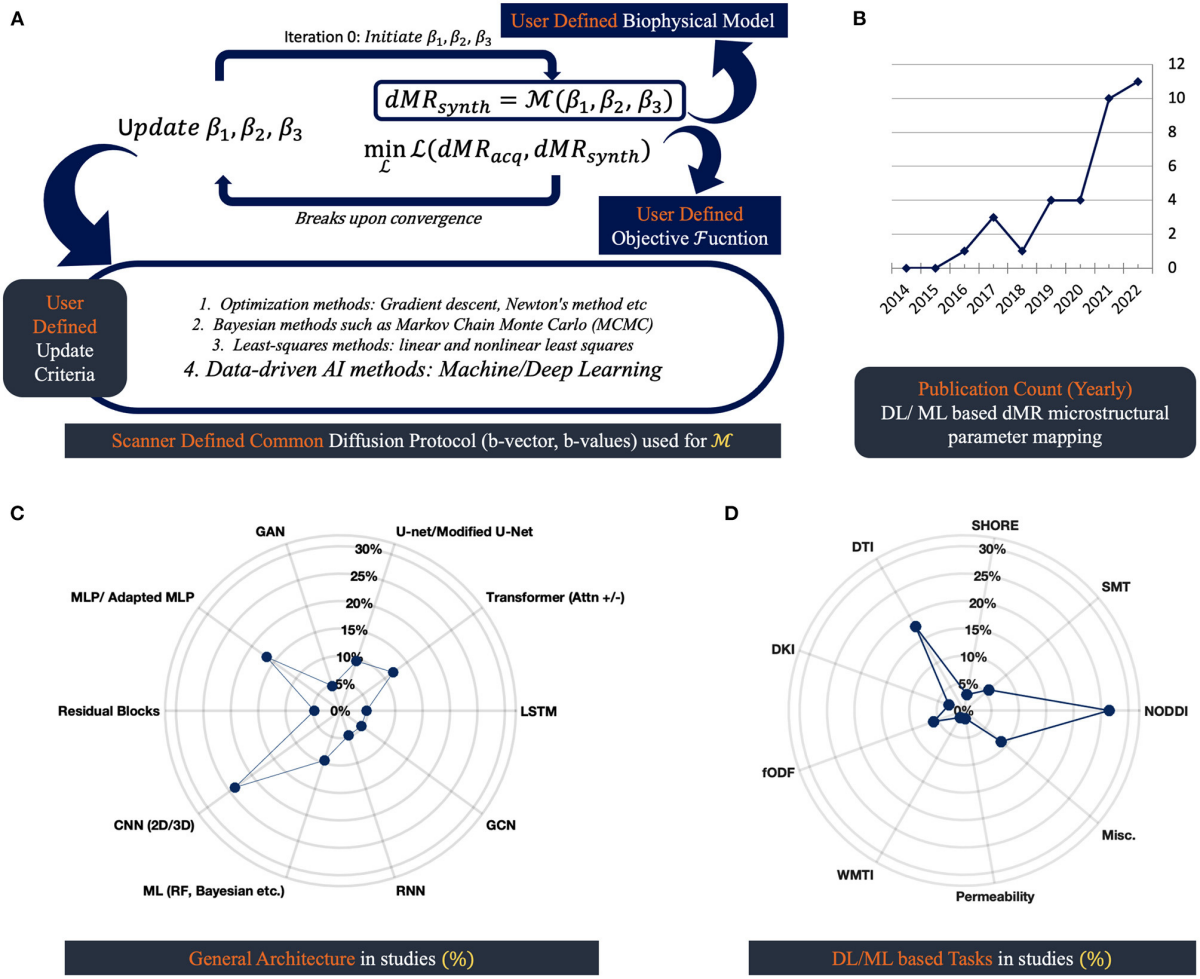
dMRI based microstructural reconstruction in human brain: schematics and progress through AI: **(A)** general schematics of microstructural reconstruction using biophysical model; **(B)** displays the number of AI approaches proposed over the last decade; **(C)** shows type of architectures these approaches use; **(D)** shows the biophysical models these AI approaches are applied to.

Overparameterization in biophysical models is necessary but results in a loss of uniqueness of the DW signals and estimated tissue microparameters, leading to higher ill-posedness in solving inverse problems (9, 40, 49, 50). A general schematic framework is illustrated in Figure 1A for parametrizing and solving inverse problems devised with a biophysical model. Like most other problems in practice, figuring out these parameters is often ill-posed by nature, which means that for a voxel, there can be multiple sets of plausible parameters that are able to explain the DWI signals. These ill-posed cases can be addressed with further contrast or geometrical priors available from the data. The ML/DL techniques can also be used to generate additional priors to solve such problems.

Once we have robust priors that distinguish the relevant features, optimization techniques need to be chosen empirically based on performance, and this is subject to validation. A fitting strategy refers to the methods and techniques used to train a model, such as gradient descent or genetic algorithms. The choice of optimization and fitting strategies can have a significant impact on the performance of a model and the ability to find a globally optimal solution (25). The following approaches—numerical analysis, Monte Carlo, animal models, phantoms, tissue fixation experiments, etc.—are commonly used to validate these models (50). The validation modes can often lead to good training data sources in DL/ML practice (30, 51).

## 3. AI in dMRI microstructure estimation

AI algorithms have been applied widely throughout the dMRI field, including signal reconstruction, denoising, detection and removal of artifacts, segmentation, co-registration, spatial and angular super-resolution of the dMRI signal, and tissue microstructure modeling (18, 21, 24, 30, 52–54). Table 1 identifies such approaches (Block B-D) and lists relevant biophysical models on which DL/ML approaches have been used (Block A). Details are provided in Supplementary Tables S1–S4.

### 3.1. Maximum likelihood frameworks for AI

Figure 1A depicts the three basic components of extracting microstructure from scanner-derived dMRI data. Voxel-wise processing of DW data requires us to have a mathematical representation or biophysical model, an optimization algorithm, and an objective function that fundamentally designs the goal of the optimizer. Before the advent of DL tools, gradient descent, Newton's method, and the Levenberg-Marquardt algorithm were popular for solving inverse problems (55). These algorithms have been used with objective functions that closely mimic the noise distribution of the data. Gaussian or Rician noise is a common find in MRI, so the optimizers are tasked with generally maximizing the log-likelihood given measured DW data for such noise distributions. Also, in some cases, the problems are reformatted in a sparse dictionary framework (56). Common practice involves adding regularization terms with objective functions in cases where problems are heavily ill-posed. To stabilize the ill-posedness of the problems, Lasso (L1), Ridge (L2), Tikhonov, etc., regularizing frameworks are used (34, 56, 57). Limitations of the maximum likelihood estimation (MLE) frameworks often involved the solution stopping at local minima, which heavily depended on the set of parameters used for initializing the biophysical model (11, 58). That's why grid-searching approaches are common to get a good starting point for the algorithms. Also, these approaches are computationally heavy (49, 59). However, a sparse dictionary representation of such a model is shown to reduce computational redundancy at the cost of accuracy (56).

Imaging reconstruction practices involving optimizers are now heavily shifting toward DL/ML. A major advantage of these approaches is generalization, but this major advantage doesn't come bias-free (60), and promising workarounds help to overcome these limitations. Data engineering is a promising approach that minimizes training-data bias by increasing data priors either by leveraging problem geometry or employing different available modalities that work as a prior. This can be easily termed “prior regularization” since it pushes the solution out of local minima. The objective values have been shown to improve and reduce bias when the starting points of the MLE are determined through an adapted multiple-layer perceptron (MLP) (30, 58).

The usage of DL/ML architectures is on the rise (Figure 1B). Different proposed approaches to data-driven strategies lack standardization in nomenclature and make it hard to track the underlying generic architecture in use. Figures 1C, D summarizes the general architectures in practice that are included in this study in a quantifiable manner. It is crucial to monitor the underlying architectures, as they come with specific limitations and pose distinctive biases. For example, it is common for convolutional neural network (CNN), U-net, and generative adversarial networks (GAN) architectures to hallucinate complex structures that might be clinically misleading (13, 33, 34, 61, 62). And for general MLP, overparameterization with noise generalizes the outcome as the mean of the training data (60).

We focused on providing the reader with a transparent view of the generic form of the algorithms in use through Table 1, with further details available in Supplementary Tables S1–S4, hoping this would create a meaningful approach to understanding data-driven AI strategies to solve the problem of microstructure estimation. The novelty of these approaches is evidently in data engineering, hyperparameter tuning, and leveraging synthetic or data-inherent priors that closely relate to the estimated parameters.

### 3.2. Unified AI (DL/ML) strategies for biophysical models: application and advancements

Based on how the DL/ML algorithms are applied to estimate tissue microparameters from dMRI data (single- or multishell DW data), including NODDI parameters such as NDI, ODI, and f_ISO_ defined in Table 1 Block-A, we have divided them into three categories found in Table 1 (Blocks B, C, and D) and in Supplementary Tables S2–S4.

**AI agnostic to dMR q-space:** The first category of AI algorithms focuses on direct DWI signal mapping and is generally agnostic to q-space geometry or how the sampling scheme is oriented for the signal. Some of these algorithms are analogous to the Natural Language Processing (NLP) algorithms that are often used in speech data processing. Examples include recurrent neural networks (RNN), short-term long memory (LSTM), GAN, attention mechanisms, etc. (63–66). The memory/forget block in some of these architectures allows for the development of signal orientation priors that are not directly sensitive to the geometry of the sampling scheme (27). These artificially generated priors might be misleading when substantial noise is present (34), as it has been noted in the literature that with lower SNR, AI algorithms are more susceptible to training data bias (60).

**AI with active use of q-space:** The second category of algorithms is much more diverse in its use of the geometry of the q-space. An inherent property of some of the architectures in this group helps to preserve this preceding geometry information. For example, graph and spherical convolutional (GCN/SCN) approaches are used to extract features that are relevant to the geometry of the acquisition schemes (24, 29). As the geometry of the q-space is incorporated, the mapping algorithms in the first category have been shown to be used in parallel to further enhance their performance (67). Q-space dMRI regression is yet another unexplored area that has shown promising results when used with an optimized protocol and a subsampling scheme (35, 68). Further, embeddings specifically designed over q-space analogous to zonal features have been shown to map fODF using adapted MLPs (20). Thus, we believe q-space is a natural characteristic of the diffusion protocol with enough potential to exploit.

**AI and MLE integrated frameworks:** The third category of algorithms embraces the recent trend of enhancing the Maximum Likelihood framework's performances through Deep Learners. Gradient update computation and initialization are challenging areas for which MLE algorithms often become stuck in the local minima (58). The advent of DL/ML has contributed to the generalization of the gradient update framework for processing variant forms of data. Previously, signals and systems being analyzed with a forward model contributed to system-specific gradient computation either analytically or numerically, which often posed computational and tedious derivation challenges, specifically with complex biophysical models such as NODDI and SMT, and this complexity increased with new parameters introduced to the system. With that said, system-specific derivatives with a good choice of optimization framework in MLE can be more powerful to rid bias and ensure specificity, which is important clinically. As spatial networks such as CNN, U-net, and GAN-based models often contribute to hallucination and systemic bias, this is often a risky bet in clinical implementation, and this recent shift in DL-based instructions for improving MLE can help overcome such issues effectively (30, 54, 58).

### 3.3. Challenges and possible solutions for implementing AI in dMRI microstructure estimation

Despite the promising results, reliable applications of AI in dMRI microstructure estimation are still challenging. Everyday challenges to data-driven techniques are often related to over- or underfitting, non-convergence, noise, hyperparameter tuning, etc. These are frequently encountered in every form of ML/DL algorithm. Therefore, we focused on challenges that are unique to clinical dMRI and biophysical models of learning. They are listed below with possible resolutions:

- **Over/underfitting:** If validation loss was significantly higher or lower than training loss, they were an indicator of over/underfitting; the validation loss trend was expected to be slightly higher than in training (30).
  To resolve the issue, one can check for inconsistencies in the training and validation data. If the data check out, relevant hyperparameters of the optimization algorithm (e.g., learning rate, momentum, etc.) need to be tested.
- **DWI noise:** With higher b-values, we often have DW images with lower SNR. And with lower SNR, the DLs are shown to be training data biased (60).
  Possible solutions include principal component analysis (PCA), DL-based denoising (15, 17, 69, 70), or using high-resolution priors (30). PCA accounts for noise by projecting data onto dimensions with the highest variances (60), whereas DL-based approaches mostly focus on spatial learning to improve SNR. However, this may not always be suitable in a clinical context due to the risk of hallucinations. High SNR priors have been shown to reliably help in DL-based estimation (30).
- **Non-uniqueness:** Often, biophysical models have multiple solutions that are all consistent with the data.
  The non-uniqueness problem in AI architectures must be dealt with by incorporating necessary boundary conditions and additional priors (71). Regularization also addresses this issue by removing fitting noise. Neural networks often use dropout layers or apply L1/L2 regularization in their proposed objective functions to resolve this problem.
- **Clinical training data scarcity:** The amount of data available to fit a model is often limited clinically, which limits inference confidence.
  Possible solutions include *in-vivo* simulations and data augmentation (71). In dMRI, the number of clinical subjects required can be substantially reduced by reducing spatial priors in the architecture. We recommend incorporating q-space priors for better clinical relevance over spatial networks (30). Generalizing the model on q-space for dMRI also increases the training samples and helps satisfy the learning goals effectively.
- **High dimensionality:** When the number of parameters in a model is high, it can be difficult to determine the correct solution with too many plausible combinations of parameters.
  The Bayesian solution has been shown to address such problems (71). It's also possible to find embeddings that are common to high-dimensional parameters (28). The so-called “embeddings” are mappings of lower-dimensional data to higher dimensions through the deep layers. In this context, AI-MLE architectures are another way to resolve this problem (54, 72). AI-MLE architectures can leverage Rician noise-based likelihood functions and optimize quickly and reliably.
- **Incorporating model limitations:** Some models have limitations on the range of parameter values that they can accurately represent, making it difficult to fit them.
  Conditions can be imposed on such limitations, followed by normalization (27, 51).
- **Multi-site data:** Some data may have non-stationary properties, meaning that the statistical properties of the data may change over time. This is commonly found in clinical dMRI as a multi-scanner harmonization problem.
  DL harmonization is seen as a potential solution (73). Data harmonization is a technique that aims to statistically standardize data from different sources so that they can be used together. Moreover, AI-MLE variants such as dtiRIM (54), which work by estimating learning gradient, have shown to be generalizable in simulation with different diffusion protocols in clinical and research settings.
- **Uncertainty quantification:** DL/ML are advanced statistical tools that should practice uncertainty quantification, a practice that is scarcely observed in the field (74). After all, only by recognizing the extent of one's ignorance can one truly grasp the depth of his/her understanding, which is true for both natural and artificial intelligence.

## 4. Future perspectives on AI in dMRI microstructure estimation

The future role of AI is highly dependent on the clinical reliability and feasibility of proposed algorithms in practice. Feasibility challenges include the number of required subjects needed for training the AI models, incompleteness and ill-posedness in the training domain, dMRI protocol complexity and scan time, and multi-scanner dataset handling. Reliability challenges include a lack of explicit instructions on extrapolation, uncertainty quantification of the proposed models, biophysical models, and inverse solvers with higher specificity, which are ideally desired but often pose limiting assumptions, etc. Since active use of q-space geometry ensures inherent priors for inference and MLE warrants likelihood with a known noise distribution framework, adapting these practices with AI is highly likely to ensure the feasibility and reliability challenges mentioned above. On the other hand, the use of spatial networks in dMRI would be more prone to deviate from clinical relevance, as it is known to be biased on spatial relevance for inference. Clinical validation has become necessary to further probe into these architectures.

## 5. Conclusion

This article has described an overview of the AI methods used for microstructure estimation/enhancement through dMRI data. The growth of AI is a captivating development, beginning with neural networks and advancing into sophisticated DL structures, allowing us to investigate brain microstructures in millimeter-scale clinical-dMRI data. While concerns regarding bias in training data exist, certain architectures have demonstrated solutions that align with diffusion biophysical models. Despite this progress, there are obstacles to overcome, particularly in clinical validation, liability for widespread adoption, and ethical and legal concerns.

## Author contributions

AF: study concept and design and writing the original draft of the manuscript. MD: revision of the manuscript for intellectual content. GS: revision of the manuscript for intellectual content and funding acquisition. MU: study concept and design, critical revision of the manuscript, and supervision. All authors read and approved the final version of the manuscript.
