# Supplementary material for: Artificial intelligence for diffusion MRI-based tissue microstructure estimation in the human brain: an overview
Source: Front Neurol. 2023 Apr 21;14:1168833. doi: 10.3389/fneur.2023.1168833 (PMC10160660; doi:10.3389/fneur.2023.1168833)
Supplement: Supplementary file 1 [file Table_1.docx]

| **Supplementary Table S1** | | | |
| --- | --- | --- | --- |
| **Block A: Biophysical Models representing Microstructural features** | | | |
| **Biophysical Models** | | **Biophysical Parameters relating Microstructural features** | **Protocol feasibility** |
| (Pasternak *et al.*, 2009) | FWE-DTI | FW, DTensor, FA, MD, AD, RD | Single/Multishell |
| (Zhang *et al.*, 2012) | NODDI | ODI, NDI, fISO | Multishell |
| (Kaden *et al.*, 2016; Kaden, Kruggel and Alexander, 2016) | Spherical Mean Technique (SMT) | SMT_FA ( $\mu$FA), SMT_MD, LDC (Longitudinal Diffusion Coefficient), TDC (Transverse DC), Anisotropy Index ($\mu$FA= LDC/TDC) | Multishell |
| (Palombo *et al.*, 2020) | SANDI (Ball, Stick, Sphere model) | f_in, f_ec, f_is, D_in, D_ec, r_s | Multishell (b-value>3000s/mm^2^) |
| (Novikov *et al.*, 2018) | Standard Model | f, Da, De_perp, De_parallel. | Multishell |
| (Ning, Westin and Rathi, 2015) | SHORE | RTOP, MSD | Multishell |

| **Supplementary Table S2** | | | | | | | |
| --- | --- | --- | --- | --- | --- | --- | --- |
| **Block B: diffusion MRI data mapping with AI (ML/DL) agnostic to q-space Geometry** | | | | | | | |
| **AI Models** | | **General architecture** | **Dataset  Total# (Train#:Test#: Validation#)** | **Loss/ Optimizer** | **Total gradient directions (b-vectors)** | **b-value (s/mm^2^)** | **Task** |
| (Zheng, Zheng, *et al.*, 2022) | AEME | LSTM | HCP Young Adult  **26** (17:2:81); HCP **10** (27:3:70) | MSE/ Adam | 60, 36, 24 | 1000, 2000 | NODDI |
| (Zheng, Sun, *et al.*, 2022) | METSC (Adapted from ViT) | Transformer (Encoder- Decoder) | HCP **26** (17:2:81); Private IVIM **24** (56:6:38) | MSE/ Adam | 60, 36, 24 | ­­1000, 2000 | NODDI |
| (Tian *et al.*, 2022) | SDnDTI | Modified U-net | HCP **20**; HCP in Aging **20** | MAE/ Adam | 186, 93, 18, 12 | 1500, 1500 | dMRI denoising |
| (Karimi and Gholipour, 2022b) | Transformer | Transformer (Attention) | HCP **200** (67:17:17), PING **20 (0:0:100),** VOGM **7 (0:0:100)** | MSE/ Adam | 88 | 1000 | DTI |
| (Karimi and Gholipour, 2022a) | ADL (Atlas powered DL) | U-net++ | Developing HCP **300** (77:0:23) | L2-norm/Adam | FA(88,12,6),ODI(300,30, 12) | FA(1000) ODI( 1000, 2600) | FA, ODI |
| (Jha *et al.*, 2022) | VRfRNet* | GAN | HCP **30** (80:3:17) | Mixed loss= adversarial+L1+total variational/ Adam | 90, 28, 15, 6 | 1000 | fODF |
| (Reisert *et al.*, 2017) | Bayesian | Bayesian ML | HCP **1** | Rician_Loglikelihood/NA | HCP (90) | HCP (1000.2000,3000) | Any, NODDI |
| (Ma and Peng, 2021) | IQT with Auto-Encoder | Residual Network | HCP **72** (N/A) | MSE+VGG Loss/ Adam | N/A | 1000 | DTI Super-resolution |
| (Tian *et al.*, 2021) | SRDTI | CNN (3D) | HCP **200** (72:18:10) | L2 Loss/ Adam | 90 | 1000 | DTI Super resolution |
| (Qin *et al.*, 2021) | Multimodal SRqDL | CNN (3D) | HCP-MGH **32** (16:0:84) | MSE/ Adam | 512 | 1000, 3000, 5000, 10000 | NODDI, SMT Super-resolution |
| (Qin *et al.*, 2021) | Super resolved q-space DL (SRqDL) | CNN | HCP-WuMinn **25** (20:0:80); HCP-MGH | MSE/ Adam | 270, 36 | 1000, 2000, 3000 | NODDI |
| (Li *et al.*, 2021) | SuperDTI | U-Net | HCP Young Adult **50** (60:20:20) | MSE/ Adam | 270, 36, 18, 6 | 1000, 2000, 3000 | DTI |
| (Karimi, Jaimes, *et al.*, 2021) | Fetal MRI | CNN + Residual Block | dHCP **102** (80:0:20) | MSE/Adam | 88 | 400, 500, 1000, 2600 | DTI |
| (Tian *et al.*, 2020) | DeepDTI | CNN | HCP WU-Minn-Ox | MSE/ Adam | 90 | 1000 | DTI |
| (Koppers *et al.*, 2019) | SHResNet | CNN + Residual Block | CDMRI Harmonization Challenge **10** (N/A) | MSE/ Adam+SGD | 30 | 1200 | DWI harmonization |
| (Ye, Li and Chen, 2019) | MESC-Net | LSTM | HCP **25** (20:0:80) | MSE/ Adam | 270, 90, 60 | 1000, 2000, 3000 | SMT, NODDI, SHORE |
| (Gibbons *et al.*, 2019) | CNN-NODDI | CNN (2D) | Private **48** (70:18:12) | MAE/ Adam | 128, 64, 24, 8 | 400, 1000, 2000 | NODDI, GFA |
| (Blumberg *et al.*, 2018) | Deeper IQT with RevNet | ML | HCP | RMSE/Adam | 90 | 1000 | DTI super-resolution |
| (Ye, 2017) | MEDN/ PMEDN (Dictionary based learner) | Adapted MLP | HCP **25** (20:0:80) | MSE/ Adam | 270, 60 | 1000, 2000, 3000 | NODDI |
| (Alexander *et al.*, 2017) | IQT | ML (Regression Forest) | HCP **24** (67:0:33), HCP Lifespan **26** (0:0:100), Private **13** | maximize *information gain* at nodes | HCP (270), HCP Lifespan (N/A), Monkey (N/A) | HCP (1000, 2000, 3000), HCP Lifespan (1000, 2500), Monkey (2000, 3000, 9500) | NODDI, SMT |
| (Nedjati-Gilani *et al.*, 2017) | Trained Random Forest | ML (Regression Forest) | Private **4** (N/A) | maximise the *information gain* at nodes | 23, 23, 23, 23 | 1622, 1718, 3611, 4031  (Δ 0.102, 0.412, 0.406, 0.169s) | Permeability |
| (Golkov *et al.*, 2016) | q-DL | MLP | HCP **2** (N/A); Private Datasets **12** (N/A) | MSE/ SGD | 288, 158, 75, 40, 30, 25, 12, 8 | 600, 750, 1070, 1200, 1800, 2400, 3000 | DKI, NODDI |

| **Supplementary Table S3** | | | | | | | |
| --- | --- | --- | --- | --- | --- | --- | --- |
| **Block C: AI data mapping with active use of q-space Geometry** | | | | | | | |
| **Models** | | **General architecture** | **Dataset  Total# (Train#:Test#: Validation#)** | **Loss/ Optimizer** | **Total gradient directions (b-vectors)** | **b-value (s/mm^2^)** | **Task** |
| (Diao and Jelescu, 2023) | ED-RNN | RNN based Encoder- Decoder | HCP **6** (80:10:10); Rat model of Alzheimer’s disease (Tristão Pereira et al., 2021) | MSE/ Adam | 90 | 1000, 2000 | WMTI-Watson |
| (Chen *et al.*, 2022) | HGT (based on TAGCN(Du *et al.*, 2018) + RDT) | Two different stages: GCN and Transformer (Attention) | HCP **21** (48:5:48) | MSE/ Adam | 60, 30 | 1000, 2000 | NODDI |
| (Faiyaz, Uddin and Schifitto, 2022) | HemiHex-MLP | Adapted MLP | Quad 22 Challenge Data **160** (2:3:95) | MSE/ SGD, Adam, RMSprop | 61, 21 | 1000 | DTI |
| (Sedlar *et al.*, 2021) | Spherical CNN** | CNN | HCP **50** (60:20:20) | MSE/ Adam | 270 | 1000, 2000, 3000 | NODDI Super angular resolution |
| (Nath *et al.*, 2021) | Bottleneck DL* Adapted SHResNet and M-heads(Lee *et al.*, 2015; Koppers *et al.*, 2019) | CNN + Residual Block | HCP **89** (45:27:28) | MSE/ RMSprop | 270, 90 | 1000, 2000, 3000 | DTI, Ball & Stick, IVIM, SMT, NODDI |
| (Karimi, Vasung, *et al.*, 2021) | q-space feature-based MLP | MLP | dHCP **95** (79:0:21) | MSE/ Adam | 88 | 1000 | fODF |
| (Ren *et al.*, 2021) | q-space conditioned DWI Generator | U-Net, GAN | HCP500 **19** (47:5:47) | L1+L2 Loss/ Adam | [253, 270] | 1000, 2000, 3000 | NODDI, SHORE, DKI, fODF |
| (Chen *et al.*, 2020) | GCNN | GCN | BCP **13** (38:0:62) | L1 Loss/ Adam | 144 | 500, 1000, 1500, 2000, 2500, 3000 | NODDI |
| (Lin *et al.*, 2019) | CNN* | CNN (3D) | HCP **30** (72:8:20) | MSE/ Adam | 270,120, 95, 90, 85, 75, 65, 60, 55, 45, 40, 35, 30, 25, 20 | 1000, 2000, 3000 | fODF |

| **Supplementary Table S4** | | | | | | | |
| --- | --- | --- | --- | --- | --- | --- | --- |
| **Block D: Models leveraging AI and Maximum Likelihood Estimation (MLE) frameworks** | | | | | | | |
| **Recent trends in AI models** | | **AI-MLE Integrated architecture** | **Dataset  Total# (Train#:Test#: Validation#)** | **Loss/ Optimizer** | **Total gradient directions (b-vectors)** | **b-value (s/mm^2^)** | **Task** |
| (Faiyaz *et al.*, 2021, 2022) | DL prior NODDI (DLpN) | Modified MLP initializes MLE | HCP **8** (23:3:75); Private CSVD **16** (11:1:88) | MSE+Rician-Loglikelihood/ Adam | 270, 180, 90 | 1000, 2000, 3000 | Single Shell NODDI |
| (Ting *et al.*, 2022) | DL-MLE | Modified MLP initializes MLE | Private **5** (N/A) | MSE+Rician- Loglikelihood/ (N/A) | 90 | 711, 2855 | NODDI |
| (Sabidussi *et al.*, 2023) | dtiRIM | Modified RNN calculating MLE gradient update | NFG Simulation; | Modified MSE/ Adam | 90, [68, 7] | 1000 | DTI |

**References**

Alexander, D.C. *et al.* (2017) ‘Image quality transfer and applications in diffusion MRI’, *NeuroImage*, 152, pp. 283–298. Available at: https://doi.org/10.1016/j.neuroimage.2017.02.089.

Blumberg, S.B. *et al.* (2018) ‘Deeper Image Quality Transfer: Training Low-Memory Neural Networks for 3D Images’, in A.F. Frangi et al. (eds) *Medical Image Computing and Computer Assisted Intervention – MICCAI 2018*. Cham: Springer International Publishing (Lecture Notes in Computer Science), pp. 118–125. Available at: https://doi.org/10.1007/978-3-030-00928-1_14.

Chen, G. *et al.* (2020) ‘Estimating Tissue Microstructure with Undersampled Diffusion Data via Graph Convolutional Neural Networks’, in A.L. Martel et al. (eds) *Medical Image Computing and Computer Assisted Intervention – MICCAI 2020*. Cham: Springer International Publishing (Lecture Notes in Computer Science), pp. 280–290. Available at: https://doi.org/10.1007/978-3-030-59728-3_28.

Chen, G. *et al.* (2022) ‘Hybrid Graph Transformer for Tissue Microstructure Estimation with Undersampled Diffusion MRI Data’, in L. Wang et al. (eds) *Medical Image Computing and Computer Assisted Intervention – MICCAI 2022*. Cham: Springer Nature Switzerland (Lecture Notes in Computer Science), pp. 113–122. Available at: https://doi.org/10.1007/978-3-031-16431-6_11.

Diao, Y. and Jelescu, I. (2023) ‘Parameter estimation for WMTI-Watson model of white matter using encoder–decoder recurrent neural network’, *Magnetic Resonance in Medicine*, 89(3), pp. 1193–1206. Available at: https://doi.org/10.1002/mrm.29495.

Du, J. *et al.* (2018) ‘Topology Adaptive Graph Convolutional Networks’. arXiv. Available at: http://arxiv.org/abs/1710.10370 (Accessed: 25 January 2023).

Faiyaz, A. *et al.* (2021) ‘Single-Shell NODDI Using Dictionary Learner Estimated Isotropic Volume Fraction’. arXiv. Available at: https://doi.org/10.48550/arXiv.2102.02772.

Faiyaz, A. *et al.* (2022) ‘Single-shell NODDI using dictionary-learner-estimated isotropic volume fraction’, *NMR in Biomedicine*, 35(2), p. e4628. Available at: https://doi.org/10.1002/nbm.4628.

Faiyaz, A., Uddin, M.N. and Schifitto, G. (2022) ‘Angular upsampling in diffusion MRI using contextual HemiHex sub-sampling in q-space’. arXiv. Available at: http://arxiv.org/abs/2211.00240 (Accessed: 19 January 2023).

Gibbons, E.K. *et al.* (2019) ‘Simultaneous NODDI and GFA parameter map generation from subsampled q-space imaging using deep learning’, *Magnetic Resonance in Medicine*, 81(4), pp. 2399–2411. Available at: https://doi.org/10.1002/mrm.27568.

Golkov, V. *et al.* (2016) ‘q-Space Deep Learning: Twelve-Fold Shorter and Model-Free Diffusion MRI Scans’, *IEEE Transactions on Medical Imaging*, 35(5), pp. 1344–1351. Available at: https://doi.org/10.1109/TMI.2016.2551324.

Jha, R.R. *et al.* (2022) ‘VRfRNet: Volumetric ROI fODF reconstruction network for estimation of multi-tissue constrained spherical deconvolution with only single shell dMRI’, *Magnetic Resonance Imaging*, 90, pp. 1–16. Available at: https://doi.org/10.1016/j.mri.2022.03.004.

Kaden, E. *et al.* (2016) ‘Multi-compartment microscopic diffusion imaging’, *NeuroImage*, 139, pp. 346–359. Available at: https://doi.org/10.1016/j.neuroimage.2016.06.002.

Kaden, E., Kruggel, F. and Alexander, D.C. (2016) ‘Quantitative mapping of the per-axon diffusion coefficients in brain white matter’, *Magnetic Resonance in Medicine*, 75(4), pp. 1752–1763. Available at: https://doi.org/10.1002/mrm.25734.

Karimi, D., Vasung, L., *et al.* (2021) ‘A machine learning-based method for estimating the number and orientations of major fascicles in diffusion-weighted magnetic resonance imaging’, *Medical Image Analysis*, 72, p. 102129. Available at: https://doi.org/10.1016/j.media.2021.102129.

Karimi, D., Jaimes, C., *et al.* (2021) ‘Deep learning-based parameter estimation in fetal diffusion-weighted MRI’, *NeuroImage*, 243, p. 118482. Available at: https://doi.org/10.1016/j.neuroimage.2021.118482.

Karimi, D. and Gholipour, A. (2022a) ‘Atlas-powered deep learning (ADL) -- application to diffusion weighted MRI’. arXiv. Available at: http://arxiv.org/abs/2205.03210 (Accessed: 2 January 2023).

Karimi, D. and Gholipour, A. (2022b) ‘Diffusion tensor estimation with transformer neural networks’, *Artificial Intelligence in Medicine*, 130, p. 102330. Available at: https://doi.org/10.1016/j.artmed.2022.102330.

Koppers, S. *et al.* (2019) ‘Spherical Harmonic Residual Network for Diffusion Signal Harmonization’. Edited by E. Bonet-Carne et al., pp. 173–182. Available at: https://doi.org/10.1007/978-3-030-05831-9_14.

Lee, S. *et al.* (2015) ‘Why M Heads are Better than One: Training a Diverse Ensemble of Deep Networks’. arXiv. Available at: https://doi.org/10.48550/arXiv.1511.06314.

Li, H. *et al.* (2021) ‘SuperDTI: Ultrafast DTI and fiber tractography with deep learning’, *Magnetic Resonance in Medicine*, 86(6), pp. 3334–3347. Available at: https://doi.org/10.1002/mrm.28937.

Lin, Z. *et al.* (2019) ‘Fast learning of fiber orientation distribution function for MR tractography using convolutional neural network’, *Medical Physics*, 46(7), pp. 3101–3116. Available at: https://doi.org/10.1002/mp.13555.

Ma, W. and Peng, L. (2021) ‘Image Quality Transfer with Auto-Encoding Applied to dMRI Super-Resolution’, in *2021 4th International Conference on Advanced Electronic Materials, Computers and Software Engineering (AEMCSE)*. *2021 4th International Conference on Advanced Electronic Materials, Computers and Software Engineering (AEMCSE)*, pp. 828–831. Available at: https://doi.org/10.1109/AEMCSE51986.2021.00169.

Nath, V. *et al.* (2021) ‘DW-MRI Microstructure Model of Models Captured Via Single-Shell Bottleneck Deep Learning’, in N. Gyori et al. (eds) *Computational Diffusion MRI*. Cham: Springer International Publishing (Mathematics and Visualization), pp. 147–157. Available at: https://doi.org/10.1007/978-3-030-73018-5_12.

Nedjati-Gilani, G.L. *et al.* (2017) ‘Machine learning based compartment models with permeability for white matter microstructure imaging’, *NeuroImage*, 150, pp. 119–135. Available at: https://doi.org/10.1016/j.neuroimage.2017.02.013.

Ning, L., Westin, C.-F. and Rathi, Y. (2015) ‘Estimating diffusion propagator and its moments using directional radial basis functions’, *IEEE transactions on medical imaging*, 34(10), pp. 2058–2078. Available at: https://doi.org/10.1109/TMI.2015.2418674.

Novikov, D.S. *et al.* (2018) ‘Rotationally-invariant mapping of scalar and orientational metrics of neuronal microstructure with diffusion MRI’, *NeuroImage*, 174, pp. 518–538. Available at: https://doi.org/10.1016/j.neuroimage.2018.03.006.

Palombo, M. *et al.* (2020) ‘SANDI: A compartment-based model for non-invasive apparent soma and neurite imaging by diffusion MRI’, *NeuroImage*, 215, p. 116835. Available at: https://doi.org/10.1016/j.neuroimage.2020.116835.

Pasternak, O. *et al.* (2009) ‘Free water elimination and mapping from diffusion MRI’, *Magnetic Resonance in Medicine*, 62(3), pp. 717–730. Available at: https://doi.org/10.1002/mrm.22055.

Qin, Y. *et al.* (2021) ‘Multimodal super-resolved q-space deep learning’, *Medical Image Analysis*, 71, p. 102085. Available at: https://doi.org/10.1016/j.media.2021.102085.

Reisert, M. *et al.* (2017) ‘Disentangling micro from mesostructure by diffusion MRI: A Bayesian approach’, *NeuroImage*, 147, pp. 964–975. Available at: https://doi.org/10.1016/j.neuroimage.2016.09.058.

Ren, M. *et al.* (2021) ‘Q-space Conditioned Translation Networks for Directional Synthesis of Diffusion Weighted Images from Multi-modal Structural MRI’, in M. de Bruijne et al. (eds) *Medical Image Computing and Computer Assisted Intervention – MICCAI 2021*. Cham: Springer International Publishing (Lecture Notes in Computer Science), pp. 530–540. Available at: https://doi.org/10.1007/978-3-030-87234-2_50.

Sabidussi, E.R. *et al.* (2023) ‘dtiRIM: A generalisable deep learning method for Diffusion Tensor Imaging’, *NeuroImage*, p. 119900. Available at: https://doi.org/10.1016/j.neuroimage.2023.119900.

Sedlar, S. *et al.* (2021) ‘A spherical convolutional neural network for white matter structure imaging via dMRI’, in M. de Bruijne et al. (eds) *MICCAI 2021 - 24th International Conference on Medical Image Computing and Computer Assisted Intervention*. Strasbourg / Virtual, France (24th Medical Image Computing and Computer Assisted Intervention - MICCAI 2021, Part III), p. Pages 529-539. Available at: https://doi.org/10.1007/978-3-030-87199-4_50.

Tian, Q. *et al.* (2020) ‘DeepDTI: High-fidelity six-direction diffusion tensor imaging using deep learning’, *NeuroImage*, 219, p. 117017. Available at: https://doi.org/10.1016/j.neuroimage.2020.117017.

Tian, Q. *et al.* (2021) ‘SRDTI: Deep learning-based super-resolution for diffusion tensor MRI’. arXiv. Available at: https://doi.org/10.48550/arXiv.2102.09069.

Tian, Q. *et al.* (2022) ‘SDnDTI: Self-supervised deep learning-based denoising for diffusion tensor MRI’, *NeuroImage*, 253, p. 119033. Available at: https://doi.org/10.1016/j.neuroimage.2022.119033.

Ting, G. *et al.* (2022) ‘Deep-learning-informed parameter estimation improves reliability of spinal cord diffusion MRI’, in *Proc. Intl. Soc. Mag. Reson. Med. 30*. *Joint Annual Meeting ISMRM-ESMRMB ISMRT 31st Annual Meeting*, UK. Available at: https://ismrm-esmrmb-ismrt2022.us3.pathable.com/meetings/virtual/5LdckoF9zKbwgm56W.

Ye, C. (2017) ‘Tissue microstructure estimation using a deep network inspired by a dictionary-based framework’, *Medical Image Analysis*, 42, pp. 288–299. Available at: https://doi.org/10.1016/j.media.2017.09.001.

Ye, C., Li, X. and Chen, J. (2019) *A deep network for tissue microstructure estimation using modified LSTM units*. Available at: https://doi.org/10.1016/j.media.2019.04.006.

Zhang, H. *et al.* (2012) ‘NODDI: Practical in vivo neurite orientation dispersion and density imaging of the human brain’, *NeuroImage*, 61(4), pp. 1000–1016. Available at: https://doi.org/10.1016/j.neuroimage.2012.03.072.

Zheng, T., Sun, C., *et al.* (2022) ‘A microstructure estimation Transformer inspired by sparse representation for diffusion MRI’. arXiv. Available at: http://arxiv.org/abs/2205.06450 (Accessed: 2 January 2023).

Zheng, T., Zheng, W., *et al.* (2022) ‘An Adaptive Network with Extragradient for Diffusion MRI-Based Microstructure Estimation’, in L. Wang et al. (eds) *Medical Image Computing and Computer Assisted Intervention – MICCAI 2022*. Cham: Springer Nature Switzerland (Lecture Notes in Computer Science), pp. 153–162. Available at: https://doi.org/10.1007/978-3-031-16431-6_15.
